# CircRNA and miRNA expression analysis in livers of mice with *Toxoplasma gondii* infection

**DOI:** 10.3389/fcimb.2022.1037586

**Published:** 2022-10-27

**Authors:** Yang Zou, Jin-Xin Meng, Xin-Yu Wei, Xiao-Yi Gu, Chao Chen, Hong-Li Geng, Li-Hua Yang, Xiao-Xuan Zhang, Hong-Wei Cao

**Affiliations:** ^1^ School of Pharmacy, Yancheng Teachers University, Yancheng, China; ^2^ College of Life Sciences, Changchun Sci-Tech University, Changchun, China; ^3^ State Key Laboratory of Veterinary Etiological Biology, Key Laboratory of Veterinary Parasitology of Gansu Province, Lanzhou Veterinary Research Institute, Chinese Academy of Agricultural Sciences, Lanzhou, China; ^4^ College of Veterinary Medicine, Qingdao Agricultural University, Qingdao, China; ^5^ College of Animal Science and Veterinary Medicine, Heilongjiang Bayi Agricultural University, Daqing, China; ^6^ College of Animal Science and Technology, Jilin Agricultural University, Changchun, China

**Keywords:** *toxoplasma gondii*, non-coding RNAs, high-throughput RNA sequencing, liver, networks

## Abstract

Toxoplasmosis is an important zoonotic parasitic disease caused by *Toxoplasma gondii* (*T. gondii*). However, the functions of circRNAs and miRNAs in response to *T. gondii* infection in the livers of mice at acute and chronic stages remain unknown. Here, high-throughput RNA sequencing was performed for detecting the expression of circRNAs and miRNAs in livers of mice infected with 20 *T. gondii* cysts at the acute and chronic stages, in order to understand the potential molecular mechanisms underlying hepatic toxoplasmosis. Overall, 265 and 97 differentially expressed (DE) circRNAs were found in livers at the acute and chronic infection stages in comparison with controls, respectively. In addition, 171 and 77 DEmiRNAs were found in livers at the acute and chronic infection stages, respectively. Functional annotation showed that some immunity-related Gene ontology terms, such as “positive regulation of cytokine production”, “regulation of T cell activation”, and “immune receptor activity”, were enriched at the two infection stages. Moreover, the pathways “Valine, leucine, and isoleucine degradation”, “Fatty acid metabolism”, and “Glycine, serine, and threonine metabolism” were involved in liver disease. Remarkably, DEcircRNA 6:124519352|124575359 was significantly correlated with DEmiRNAs mmu-miR-146a-5p and mmu-miR-150-5p in the network that was associated with liver immunity and pathogenesis of disease. This study revealed that the expression profiling of circRNAs in the livers was changed after *T. gondii* infection, and improved our understanding of the transcriptomic landscape of hepatic toxoplasmosis in mice.

## Introduction

Toxoplasmosis is a widespread zoonotic disease caused by *Toxoplasma gondii* (*T. gondii*) worldwide. *T. gondii* is an intracellular apicomplexan parasite that can infect almost all warm-blooded animals and humans (Tenter et al., 2000). Gamogony and oocyst can form in the epithelium of small intestine after an ingestion of *T. gondii* by definitive feline hosts. Then, the unsporulated oocysts can beare released into the intestinal lumen and excrete with feces, leading to contamination of soil and the environment (Tenter et al., 2000). The people who ingested undercooked food or water containing tissue cysts and sporulated oocysts will be infected by *T. gondii* (Dubey, 2008). The clinical manifestations of toxoplasmosis range from asymptomatic to fatal infection, including abortion, encephalitic illness, and conjunctivitis (Smith et al., 2021). *T. gondii* can attack the host organs, including livers, lymph nodes, eyes, hearts, and central nervous systems (Montoya and Liesenfeld, 2004; Stauffer et al., 2006; Balasundaram, 2010). In livers, *T. gondii* infection can cause several pathological changes, e.g. hepatitis, hepatomegaly, granuloma, and necrosis (Karasawa et al., 1981; Ortego et al., 1990; Hassan et al., 1996; Doğan et al., 2007). However, the molecular mechanisms underlying *T. gondii* infection and liver disease remain poorly understood.

Circular RNAs are one of the novel classes of endogenous noncoding RNAs that are formed by exon-scrambling (Meng et al., 2017). With the development of RNA sequencing (RNA-seq) technology, the abundance, diversity, and dynamic expression patterns of circRNAs in various organisms have been clarified (Conn et al., 2015). CircRNAs can not only antagonize the activity of miRNA through a sponge-like mechanism but also regulate gene expression at the post-transcriptional level (Granados-Riveron and Aquino-Jarquin, 2016). A series of studies showed that circRNAs played roles in the pathological processes of liver disease (Yao et al., 2017; Tang et al., 2020). The circRNAs are used as prognostic biomarkers, owing to remarkably stable characteristics (Lei et al., 2019). In addition, circRNAs are a potential drug target for diseases (Dhamija and Menon, 2018; Zhu et al., 2018). Thus, exploration of circRNA function that connecting with liver disease induced by *T. gondii* infection will provide a novel perspective for hepatic disease treatment and diagnosis.

In the present study, RNA-seq was performed for identifying the expression of circRNAs and miRNAs in the livers of mice after *T. gondii* infection, in order to investigate the relationships between circRNAs and miRNAs in the *T. gondii* infected livers of mice. The simultaneous analyses of the differentially expressed (DE) circRNAs and DE miRNAs were conducted to investigate the relevance of the expression and circRNA-miRNA interactions. Moreover, the potential functional role was predicted. Thus, the correlation networks of circRNAs and miRNAs in the livers of mice after *T. gondii* infection improved our understanding of the transcriptomic landscape of hepatic toxoplasmosis in mice.

## Methods

### *Toxoplasma gondii*, mice and infection

*T. gondii* cysts were collected from the brains of mice that had been infected with *T. gondii* for months. In brief, the mice were sacrificed after anesthetization, and the brains were dissected and collected with a mortar for preparing tissue homogenates. The brain homogenates were rinsed with phosphate-buffered saline (PBS), and then were transferred to a 2 mL EP tube. Then, the *T. gondii* cysts in brain tissues were counted by using a dissection microscope. The 8-10 week-old female BALB/c mice (SPF) were purchased from Spaefer Biotechnology Co., Ltd. (Beijing, China). All mice were housed in cages with an independent ventilation system under a 12-h dark/light cycle, with free food and water ad libitum. The mice (n = 12) were randomly divided into three groups: acute infection group (n = 3); chronic infection group (n = 3); and control group (n=6). In the infection groups, each mouse was infected with 20 *T. gondii* cysts. In the control group, the mice were treated with PBS. A previous study showed the timing of acute and chronic infection stages in mice infected with *T. gondii* (Hu et al., 2018). The mice in each group were sacrificed on day 11 (acute infection group) and 33 (chronic infection group) after infection, respectively. The successful establishment of mouse model was examined based on amplification of *T. gondii* B1 gene as described previously (Hu et al., 2018). At the mentioned time points post infection, the livers of mice in each group were dissected from each mouse. Then, the liver samples were immediately deposited in liquid nitrogen until RNA extraction.

### RNA extraction

Approximately 50 mg of liver tissue was subjected to RNA extraction using TRIZOL (Life Technologies, Carlsbad, USA). In brief, the samples were firstly homogenized by liquid nitrogen, and the 1 ml TRIZOL reagent was added to the homogenization to lyse sample. Then, the 0.2 ml of chloroform per was added and shake tubes vigorously by hand for 15 s. The sample was incubated at room temperature for 2 to 3 minutes, and then centrifuged at 11,500 *g* for 15 min at 4°C. The RNA samples remain in the aqueous phase. The aqueous phase was transferred to a new tube, and isopropanol was added to precipitate RNA. The 75% ethanol was used to wash RNA samples; Finally, RNA pellet was redissolved with the water (Chomczynski and Sacchi, 2006). The RNA degradation and contamination were detected with 1% agarose gel test. The RNA Nano 6000 Assay Kit of the Bioanalyzer 2100 system (Agilent Technologies, CA, USA) and Qubit^®^ RNA Assay Kit in Qubit^®^ 2.0 Flurometer (Life Technologies, CA, USA) were used to measure and evaluate the concentration and purity of RNA, respectively. Then, the RNA samples were stored at −80°C for a further analysis.

### Library preparation and sequencing

Approximately 5 μg of RNA sample was used for constructing the circRNA library by using NEBNext^®^ Ultra™ Directional RNALibrary Prep Kit for Illumina^®^ (NEB, USA). In brief, the First-strand cDNA was synthesized using M-MuLV Reverse Transcriptase with random hexamer primer. Then, the cDNA fragments were purified by AMPure XP system (Beckman Coulter, Beverly, USA). The cDNA was used for PCR amplification. Agilent Bioanalyzer 2100 system was employed for assessing library quality (Zhou et al., 2017). The sequencing libraries of circRNAs were performed using Illumina Hiseq 4000 platform, and the 150 bp paired-end reads were generated.

The 3 μg of RNA sample and NEBNext^®^ Multiplex Small RNA Library Prep Set for Illumina^®^ (NEB, USA) was used for generating miRNA libraries. Briefly, the first strand of cDNA of miRNA was synthesized through M-MuLV Reverse Transcriptase. Then, the LongAmp Taq 2×Master Mix, index (X) primer, and SR primer were used for PCR amplification. Finally, the library preparations were sequenced on an Illumina Hiseq 2500/2000 platform, and 50 bp single-end reads were generated.

### Identification of circRNAs and miRNAs

The raw reads of fastq format were obtained by the Custom Perl and Python scripts. The ploy-N, with 5′ adapter contaminants, without 3′ adapters, and low reads were removed. The GC content, Q20, Q30, and the error rate were performed to assess quality of the clean data. The HISAT2 v2.0.4 and bowtie2 v2.2.8 were used for building and aligning clean data with the *Mus musculus* reference genome, respectively (Langmead and Salzberg, 2012; Pertea et al., 2016). The circRNA identification was performed using find_circ (Memczak et al., 2013) and CIRI2 (Gao et al., 2015). circRNA was predicted by the intersection between the two algorithms. Moreover, the small RNA tags were mapped to obtain known miRNAs using MiRBase 20.0 (Griffiths-Jones, 2016). The novel miRNAs were predicted by using miREvo (Wen et al., 2012) and mirdeep2 (Friedlander et al., 2012). The quantification of circRNA and miRNA expression profiles were normalized by TPM (transcript per million) (Zhou et al., 2010). The differential expression analysis was performed using the DESeq R package (1.8.3) (Anders and Huber, 2010). |Log2 fold change (FC)| ≥ 1.0 and *P*-value < 0.05 were used as thresholds to identify differentially expressed transcripts.

### MiRNA target gene prediction and functional analysis

The potential target genes of DE miRNAs were predicted by a combined use of Miranda, PITA, and RNAhybrid softwares. GO enrichment analysis of the potential target genes in the livers of mice infected with *T. gondii* was conducted using the GOseq R package (Young et al., 2010). The KEGG (Kyoto encyclopedia of genes and genome) pathway functional annotation were performed by using KOBAS 3.0 software (Mao et al., 2005). *P*-value < 0.05 was considered as significant enrichment.

### Quantitative real-time PCR analysis

The DE circRNAs and DE miRNAs were chosen to verify the RNA-Seq results by using qRT-PCR. The qPCR was performed in a LightCycler480 (Roche, Basel, Switzerland) using a ChamQ SYBR qPCR Master Mix kit (Vazyme, Nanjing, China). The reaction was consisted of 40 cycles, circRNA initial degeneration at 95°C for 30 s, and template degeneration in the PCR cycle at 95°C for 10 s, and finally annealing at 60°C for 30 s. The reactive cycle of miRNA was consisted of 95°C for 30 s, then 40 cycles of 95°C for 5 s and 60°C for 34 s. The amplification was ensured by melting curve analysis in each reaction. The primers of miRNAs and circRNAs were listed in **Table 1**. L13A and U6 were used as the internal controls of circRNA and miRNA, respectively. The relative expression quantity was calculated using the 2^-ΔΔCt^ method (Livak and Schmittgen, 2001).

**Table 1:** Primers used in lncRNA and mRNA-specific qRT-PCR analysis.

RNAs	Primer	Sequence (5’ to 3’)
15:3279732|3280203 (circRNA)	Forward primer	TGGAAGCCAATATGGTAGATTTCTC
	Reverse primer	TCATTTTCTCTCCCCAACTCAGTC
17:39848416|39848682 (circNA)	Forward primer	CCCTCGTAGACACGGAAGAGC
	Reverse primer	CTTTTCTGGCCTCGCCACC
mmu-miR-1247-3p (miRNA)	Forward primer	GGAACGTCGAGACTGGAGCA
mmu-miR-339-5p (miRNA)	Forward primer	TGTCCTCCAGGAGCTCACGA
mmu-miR-379-5p (miRNA)	Forward primer	TGGTAGACTATGGAACGTAGGA
mmu-miR-146b-5p (miRNA)	Forward primer	TGAGAACTGAATTCCATAGGCTA

## Results

### Differentially expressed CircRNAs and miRNAs

**Figure 1:**
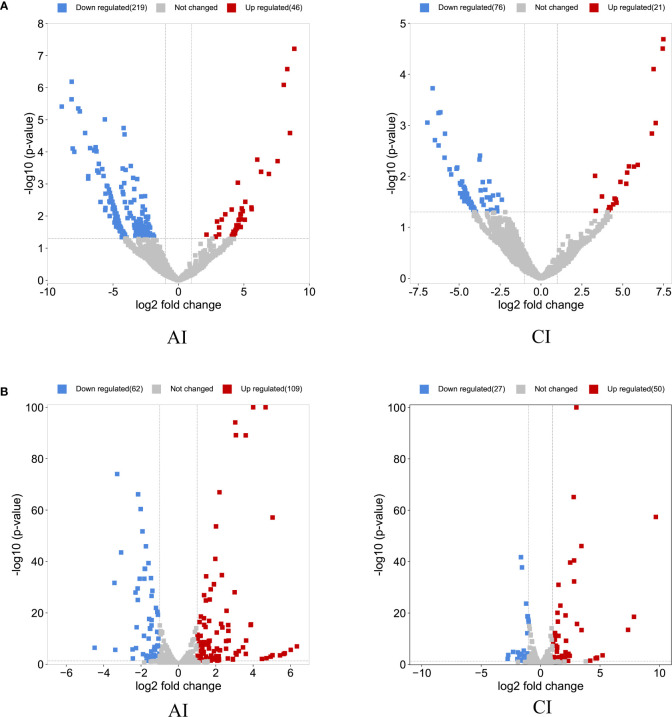
Overview of the differentially expressed (DE) circRNAs and DEmiRNAs. The volcano plots of DEcircRNAs **(A)** and DEmiRNAs **(B)** at acute infection (AI) and chronic infection (CI) stages. The horizontal-axis shows the log2 fold change, and the vertical-axis shows the -log10 p-value. The up-regulated are marked in red and the down-regulated RNAs are in blue.

**Figure 2:**
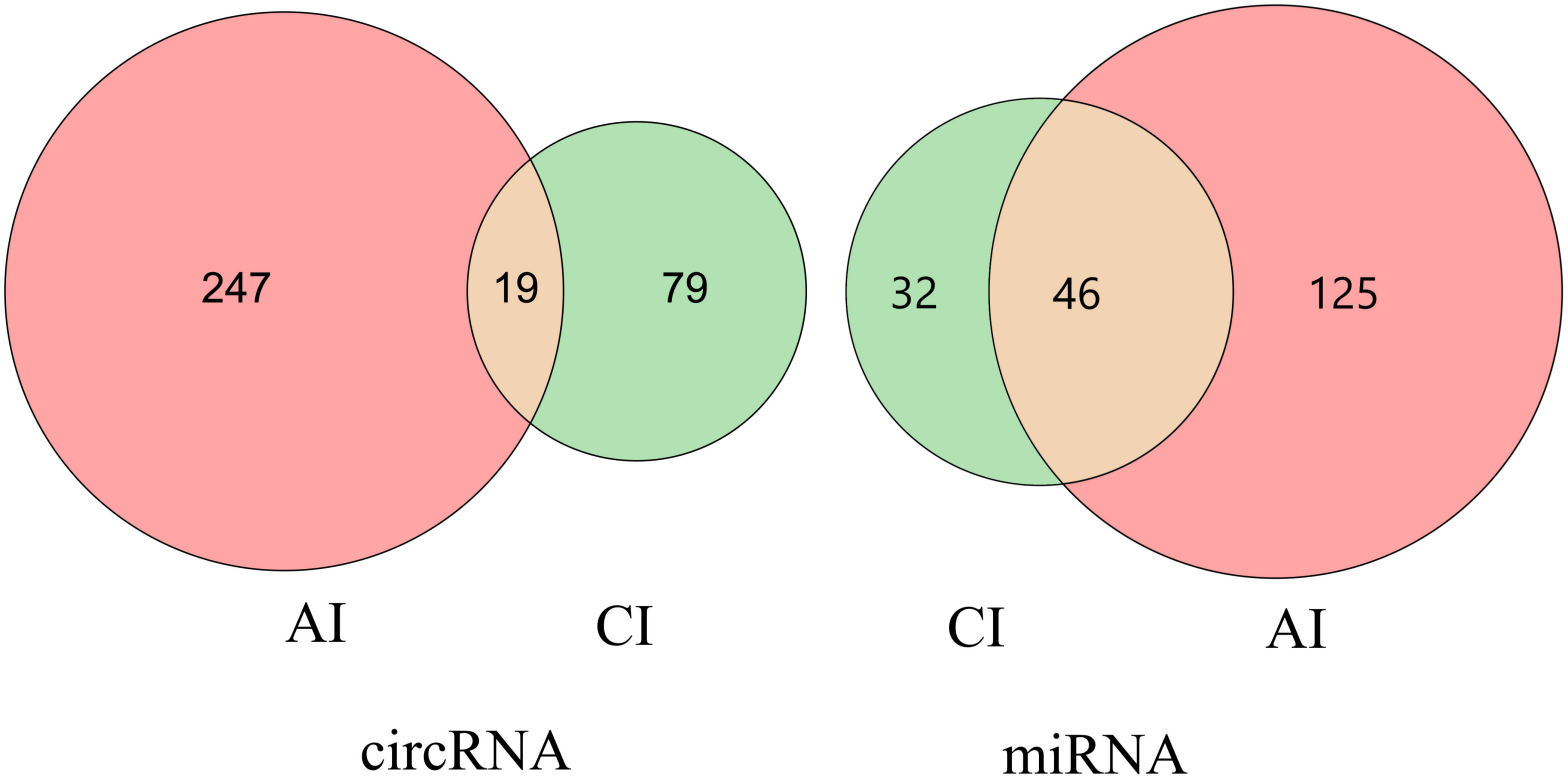
Venn diagram of the differentially expressed (DE) circRNAs and DEmiRNAs. the number of the common or unique DEcircRNAs and DEmiRNAs at two infection stages.

Compared with the control group, a total of 265 DE circRNAs and 171 DE miRNAs were identified at the acute infection stage, and 97 DE circRNAs and 77 DE miRNAs were detected in the livers at the chronic infection stage (**Figure 1** and **Supplementary Table S1**). A total of 19 circRNAs and 46 miRNAs were commonly dysregulated between the acute and chronic *T. gondii*-infected groups (**Figure 2**). Among DE transcripts, the mmu-miR-147-3p was up-regulated 32.94 folds at the acute infection stage, however, it was down-regulated to 3.66 folds at the chronic infection stage. Moreover, mmu-miR-342-3p was up-regulated 8.23 folds at the acute infection stage. Furthermore, mmu-miR-143-3p was down-regulated 4.05 folds and 2.11 folds at the acute and chronic infection stages, respectively (**Supplementary Table S1**).

### GO annotation and KEGG pathway analysis

**Figure 3:**
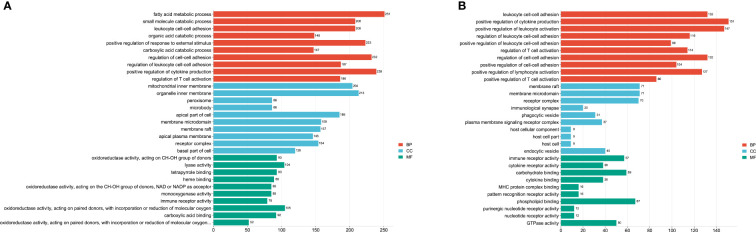
The top 30 GO terms of target genes of the differentially expressed (DE) miRNAs at acute infection **(A)** and chronic infection **(B)** stages. The vertical-axis represents the GO terms. The horizontal-axis represents numbers of target genes. The BP represents biological process, CC represents cellular component, and MF represents molecular function.

To find the potential biological associations of DE miRNAs, GO and KEGG pathway enrichment analyses for infection-associated transcripts were predicted. The top 30 GO terms were shown in **Figure 3**. Most of predicted genes were involved in the “fatty acid metabolic process”, “positive regulation of cytokine production”, “positive regulation of response to external stimulus” and “regulation of T cell activation” at the acute infection stage (**Figure 3A**). Furthermore, the biological process mainly included “leukocyte cell-cell adhesion”, “positive regulation of cytokine production”, and “positive regulation of leukocyte activation”. The cellular component included “membrane raft” and “membrane microdomain”, the molecular function included “immune receptor activity” and “phospholipid binding” at the chronic infection stage (**Figure 3B**).

**Figure 4:**
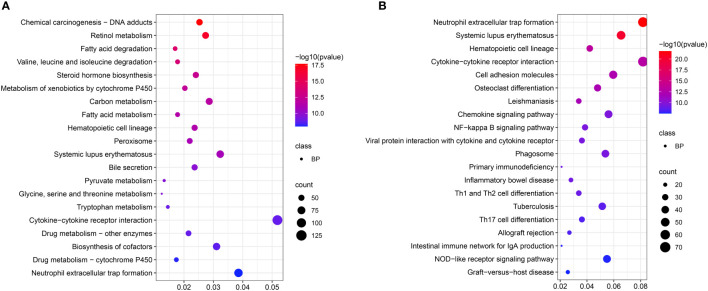
Scatter plots showing KEGG pathway of the target genes. The top 20 enriched pathways at acute infection **(A)** and chronic infection **(B)** stages, respectively. The vertical-axis represent pathways, and the horizontal-axis numbers represent the rich factor.

KEGG enrichment analysis showed that pathways mainly included “Valine, leucine, and isoleucine degradation”, “Fatty acid metabolism”, “Glycine, serine and threonine metabolism”, and “Tryptophan metabolism” (**Figure 4A**) at the acute infection stage. These results showed the hepatic metabolism affected the acute infection stage of *T. gondii*. Moreover, some pathways were related to immunity and inflammation, such as “Cytokine-cytokine receptor interaction”, “Cell adhesion molecules”, “NF-kappa B signaling pathway”, “Primary immunodeficiency”, “Inflammatory bowel disease”, “Th1 and Th2 cell differentiation”, “Th17 cell differentiation”, and “NOD-like receptor signaling pathway”. Interestingly, some pathways were related with intestinal flora, e.g. “Inflammatory bowel disease” and “Intestinal immune network for IgA production” (**Figure 4B**).

### Co-expression networks of DEcircRNAs and DEmiRNAs

**Figure 5:**
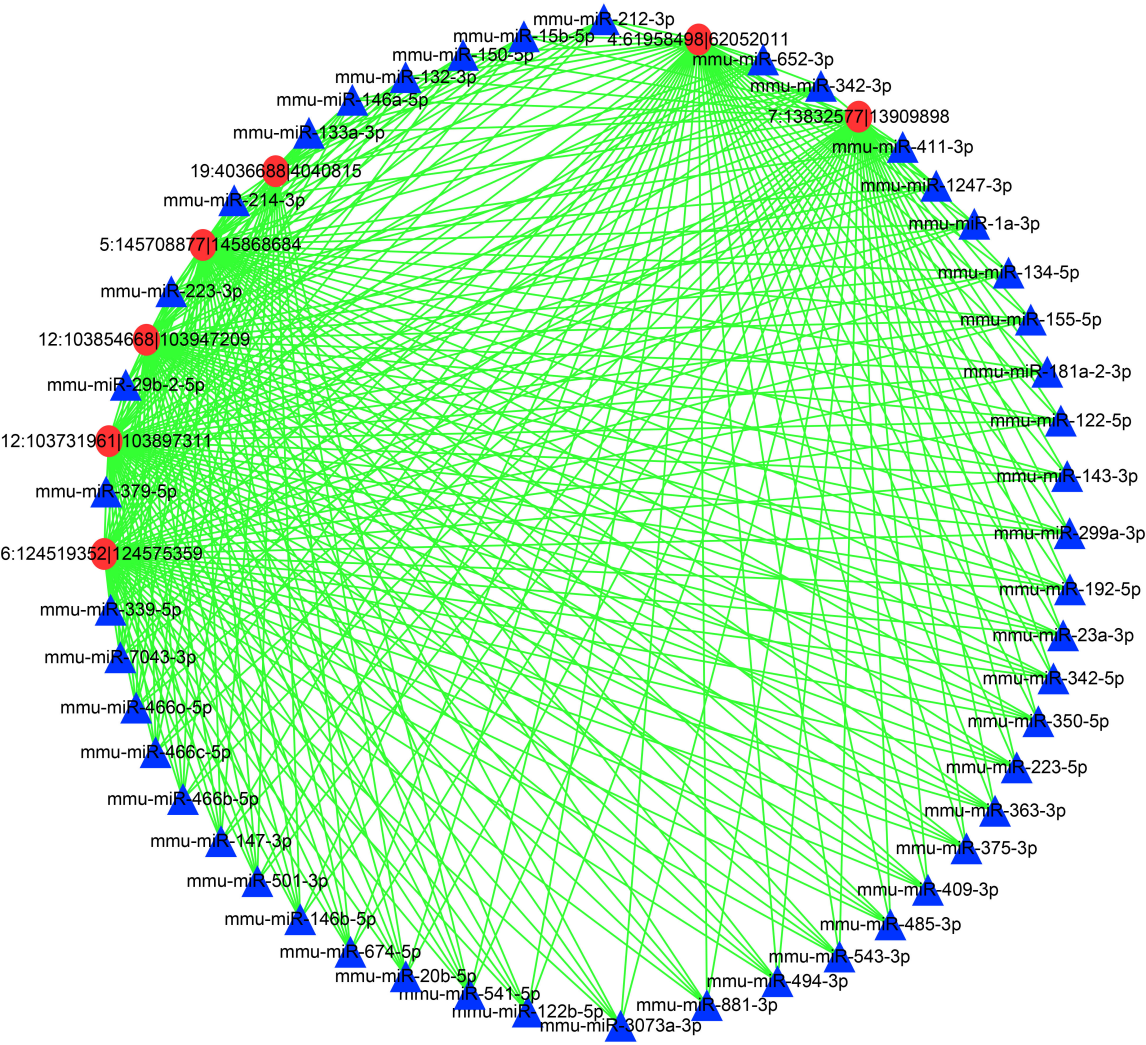
A network of the commonly DEcircRNAs with their associate DEmiRNAs. Different colors show different RNA, with red for DEcircRNAs and blue for DEmiRNAs.

To further reveal the mechanisms underlying the DEcircRNAs and DEmiRNAs in the livers during *T. gondii* infection, a network was constructed (**Figure 5** and **Supplementary Table S2**). In this network, the DEcircRNA 6:124519352|124575359 was related to DE miRNAs mmu-miR-132-3p, mmu-miR-146a-5p, mmu-miR-150-5p, and other DEmiRNAs (n = 42, **Supplementary Table S2**). Moreover, DEcircRNA 4:61958498|62052011 was associated with DEmiRNAs (mmu-miR-146a-5p) and 45 other DEmiRNAs. Moreover, DEmiRNA mmu-miR-1247-3p shared 5 DEcircRNAs, including 12:103731961|103897311, 12:103854668|103947209, 5:145708877|145868684, 4:61958498|62052011, and 7:13832577|13909898 (**Figure 5**). The networks showed that multiple miRNAs were regulated by several circRNAs at the two infection stages, thus suggesting a complex regulatory relationship between DEcircRNAs and DEmiRNAs.

### Verification of the DEcircRNAs and DEmiRNAs by qRT-PCR

To evaluate the reliability of RNA-sequence results, the expression profiles of the randomly selected DEcircRNAs and DEmiRNAs were successfully confirmed by qRT-PCR. The results obtained by RNA-Seq and qRT-PCR were consistent in the trend and magnitude of the expression (**Supplementary Figure S1**).

## Discussion

Previous omics studies have provided a wealth of resources that improved understanding of the pathogenesis of *T. gondii* (Garfoot et al., 2019; Antil et al., 2021; Menard et al., 2021; Antil et al., 2022), and many studies mainly focused on mRNAs (He et al., 2016; Zhou et al., 2016; Lutshumba et al., 2020). Recently, Zhou and colleagues revealed putative functions of miRNAs and circRNAs in brains of mice after an infection with *T. gondii* (Zhou et al., 2020). However, the expression levels of circRNAs and miRNAs specific to *T. gondii* infection in livers of mice were unclear. Thus, the present study explored the expression profiles of circRNAs and miRNAs in the livers of mice at acute and chronic stages after *T. gondii* infection by using RNA-seq technique.

MiR-147-3p (mmu-miR-147-3p) has been reported to dampen Toll-like receptor (TLR)-signaling in murine macrophages (Liu et al., 2009). It can limit excessive inflammation in the hosts response to influenza A virus infection (Preusse et al., 2017). The miR-147 plays an important role in the negative regulation of TLR/NF-κB-mediated proinflammatory cytokines, and inhibits the expression of proinflammatory cytokines (Zuo et al., 2020). In this study, the miR-147-3p was up-regulated 32.94 folds at the acute infection stage. *T. gondii* infection triggered liver inflammation at an early infection stage, and the up-regulation of miR-147-3p could limit excessive inflammation for protecting the hosts. Further protection was attenuated with increasing duration of infection, as the expression of miR-147-3p decreased to 3.66-fold during the chronic infection stage. Interestingly, miR-342-3p (up-regulated 8.23 fold) seemed to have the same effect as miR-147-3p at the acute infection stage. Overexpression of miR-342-3p can suppress inflammation response in THP-1 cells (Wang et al., 2019). Moreover, miR-342-3p was considered to be significantly relative with regulating metabolic profiles of Treg cells (Kim et al., 2020), which may explain for its role in inflammation inhibition. Moreover, miR-143-3p could participate in inflammatory pain responses in fibromyalgia patients (Jiang et al., 2015). A previous study showed that miR-143-3p might inhibit inflammatory factors’ levels through regulating the MyD88/NF-κB signaling pathway (Wang et al., 2020). In this study, the miR-143-3p (mmu-miR-143-3p) was down-regulated 4.05 folds and 2.11 folds at the acute and chronic infection stages, respectively. Thus, these findings suggest that miR-143-3p may participate in the inflammation reaction of livers during *T. gondii* infection, and the down-regulation of miR-143-3p can increase the inflammation of livers for resisting infection. In addition, these findings reveal mediation of pro-inflammatory and anti-inflammatory mechanisms in the livers in *T. gondii* pathogenesisis.

In this study, some immunity-related GO terms were enriched at the two infection stages, such as “positive regulation of cytokine production”, “regulation of T cell activation”, “leukocyte cell-cell adhesion”, “positive regulation of leukocyte activation”, and “immune receptor activity” (**Figure 3**), indicating that *T. gondii* induced the liver immunity reaction of hosts. KEGG analysis showed that a series of pathways (e.g. “Valine, leucine and isoleucine degradation”, “Fatty acid metabolism”, and “Glycine, serine and threonine metabolism”) were involved in liver disease. The reductions of valine, leucine, and isoleucine are considered to be related to hepatic encephalopathy pathogenesis, and impair liver regeneration (Fischer et al., 1975; Marchesini et al., 2003; Nakaya et al., 2007; Urata et al., 2007). Moreover, TNF-α and IL-6 activate branched-chain keto acid dehydrogenase increased valine, leucine, and isoleucine catabolism (Nawabi et al., 1990; Holecek, 1996). IL-6 and TNF-α are proinflammatory cytokines, and metabolic disturbances are strongly related to increased levels of these two cytokines (Popko et al., 2010), thus suggesting *T. gondii* causes the metabolic disturbance of the livers at the acute infection stage. The other metabolism pathways enriched in the livers at the acute infection stage indicated disorders of metabolic function of the host livers induced by *T. gondii* infection. The cell adhesion molecules are considered as targets for the bacterial pathogens in establishing intimate contact with the cells and tissues of hosts (Hauck et al., 2006). A previous study reported that targeted disruption of *SAG3* gene in *T. gondii* results in a partial decrease in host cell adhesion, and drastic reduction of virulence in mice (Dzierszinski et al., 2000). The “Cell adhesion molecules” pathway enriched at the chronic infection stage indicated potential relationships between cell adhesion molecules and *T. gondii* infection. The NF-κB family of transcription factors was closely related to the mediation of innate and adaptive immunities against infection (Caamaño et al., 1999). A previous study showed that the NF-κB2^−/−^ mice could resist *T. gondii* infection during the acute phase of toxoplasmosis, but displayed a protracted pattern of mortality during the chronic stage of infection (Franzoso et al., 1998). These findings suggest NF-κB signaling pathway is essential for *T. gondii* infection in livers. Th17 produces AMPs that prevent the dysbiosis and bacterial translocation related to the pathogenic infection through secreting IL-17, and Th17 is crucial for host survival after *T. gondii* (type II strain) infection (Cervantes-Barragan et al., 2019). Moreover, a few studies reported that IL-17 signaling played a protective role during *T. gondii* infection (Kelly et al., 2005; Moroda et al., 2017). Thus, the “Th17 cell differentiation” pathway was observed in the livers at the chronic infection stage. An early *T. gondii* infection can disrupt the resident microbial communities and induce acute inflammation in the ileum (Heimesaat et al., 2006; Raetz et al., 2013). *T. gondii* infection also induces the increment of abundance of proinflammatory proteobacteria, and decrement of beneficially bacterial communities, causing disruption of the microbial community composition that persists at the chronic infection stage (French et al., 2022). These findings support that *T. gondii* infection causes microbial imbalance. Notably, “Inflammatory bowel disease” and “Intestinal immune network for IgA production” pathways were also enriched in the livers at the chronic infection stage. These two pathways are significantly associated with microbiota (Federici et al., 2022; Tan et al., 2022; Tchitchek et al., 2022). Thus, we suspect that *T. gondii* infection could mediate hepatic metabolism, and further affecting microbial balance in mice. How this mechanism works remains to be investigated.

CircRNAs act as miRNA decoys or sponges in regulating gene expression (Panda, 2018). Thus, a correlation analysis of the expression profiles from the DEcircRNAs and DEmiRNAs predictive interactions was performed. In the network, DE miRNAs mmu-miR-146a-5p and mmu-miR-150-5p were regulated by DEcircRNA 6:124519352|124575359. miR-146a-5p plays a role in different disease contexts and acts as a negative regulator of inflammatory and immune responses (Xu et al., 2012). A previous study reported that HCV-induced increment of miR-146a-5p promoted viral infection and metabolic pathways related to pathogenesis of hepatic disease (Bandiera et al., 2016). Thus, miR-146a-5p is associated with liver immunity and pathogenesis of liver disease. The upregulation of miR-146a-5p at both two infection stages (**Supplementary Table S1**) revealed a potential role in the *T. gondii* infection induced liver disease. However, the DEcircRNA 6:124519352|124575359 mediates this process. miR-150-5p regulates target genes IL-10 and PIM1, having an anti-inflammatory effect (Neamah et al., 2019). It was also increased at both acute and chronic infection stages, and was regulated by DEcircRNA 6:124519352|124575359. This illustrates both circRNA 6:124519352|124575359 and miR-150-5p have protective effects on *T. gondii*-induced liver excessive inflammation. Moreover, mmu-miR-1247-3p was regulated by multiple circRNAs. The treatment of HCC with miR-1247-3p would increase the expression levels of IL-1β, IL-6, and IL-8, thus suggesting the proinflammatory role of miR-1247-3p (Fang et al., 2018). The DEmiRNA mmu-miR-1247-3p was regulated by DEcircRNAs 12:103731961|103897311, 12:103854668|103947209, 5:145708877|145868684, 4:61958498|62052011, and 7:13832577|13909898 in the network, indicating that these DEcircRNAs participated in liver inflammation caused by *T. gondii* infection. In this study, RNA-seq was performed to detect the differential expression profiles of circRNAs and miRNAs in the livers of mice infected with *T. gondii*. However, some limits should be addressed, including other organizations of expression profiles should be added, and the potential interaction between circRNA-miRNA and mRNA is needed to validate in the future work.

## Conclusions

This study explored the differential expression levels of circRNAs and miRNAs in the livers of mice infected with *T. gondii* at the acute and chronic stages. The functional enrichment analysis showed that many DEcirciRNAs and DE miRNAs were associated with the inflammation responses of the livers after *T. gondii* infection. Our results provided valuable data for the understanding of the circRNAs and miRNAs involved in the molecular basis of the hepatic responses to *T. gondii* infection. The functions of DEcircRNAs and DEmiRNAs in the pathogenesis of *T. gondii* infection in livers will be further verified.

## Data availability statement

The datasets presented in this study can be found in online repositories. The names of the repository/repositories and accession number(s) can be found in the article/**Supplementary Material**.

## Ethics statement

The animal study was reviewed and approved by the Animal Ethics Committee of Qingdao Agricultural University.

## Author contributions

X-XZ and H-WC conceived and designed the experiments. J-XM and X-YM performed the experiments. X-YG, X-YW, J-XM, CC and H-LC contributed reagents/materials/analysis tools. YZ analyzed the data and wrote the paper. X-YW, J-XM, L-HY, X-XZ and HW-C critically revised the manuscript. All authors read and approved the final version of the manuscript.

## Funding

This work was supported by the National Natural Science Foundation of China (Grant No. 31902238), Research Foundation for Distinguished Scholars of Qingdao Agricultural University (665-1120044).

## Conflict of interest

The authors declare that the research was conducted in the absence of any commercial or financial relationships that could be construed as a potential conflict of interest.

## Publisher’s note

All claims expressed in this article are solely those of the authors and do not necessarily represent those of their affiliated organizations, or those of the publisher, the editors and the reviewers. Any product that may be evaluated in this article, or claim that may be made by its manufacturer, is not guaranteed or endorsed by the publisher.

## Supplementary material

Supplementary Figure 1 Validation for the expression of the DEcircRNAs and DEmiRNAs by qRT-PCR at acute infection (AI) and chronic infection (CI) stages.

Supplementary Table 1 The expression profile of differentially expressed circRNAs and miRNAs in the liver at acute infection and chronic infection stages.

Supplementary Table 2 The data of DEcircRNA-DEmiRNA network analysis.
