# Supplementary figures and images for: CircRNA and miRNA expression analysis in livers of mice with Toxoplasma gondii infection
Source: Front Cell Infect Microbiol. 2022 Oct 27;12:1037586. doi: 10.3389/fcimb.2022.1037586 (PMC9646959; doi:10.3389/fcimb.2022.1037586)

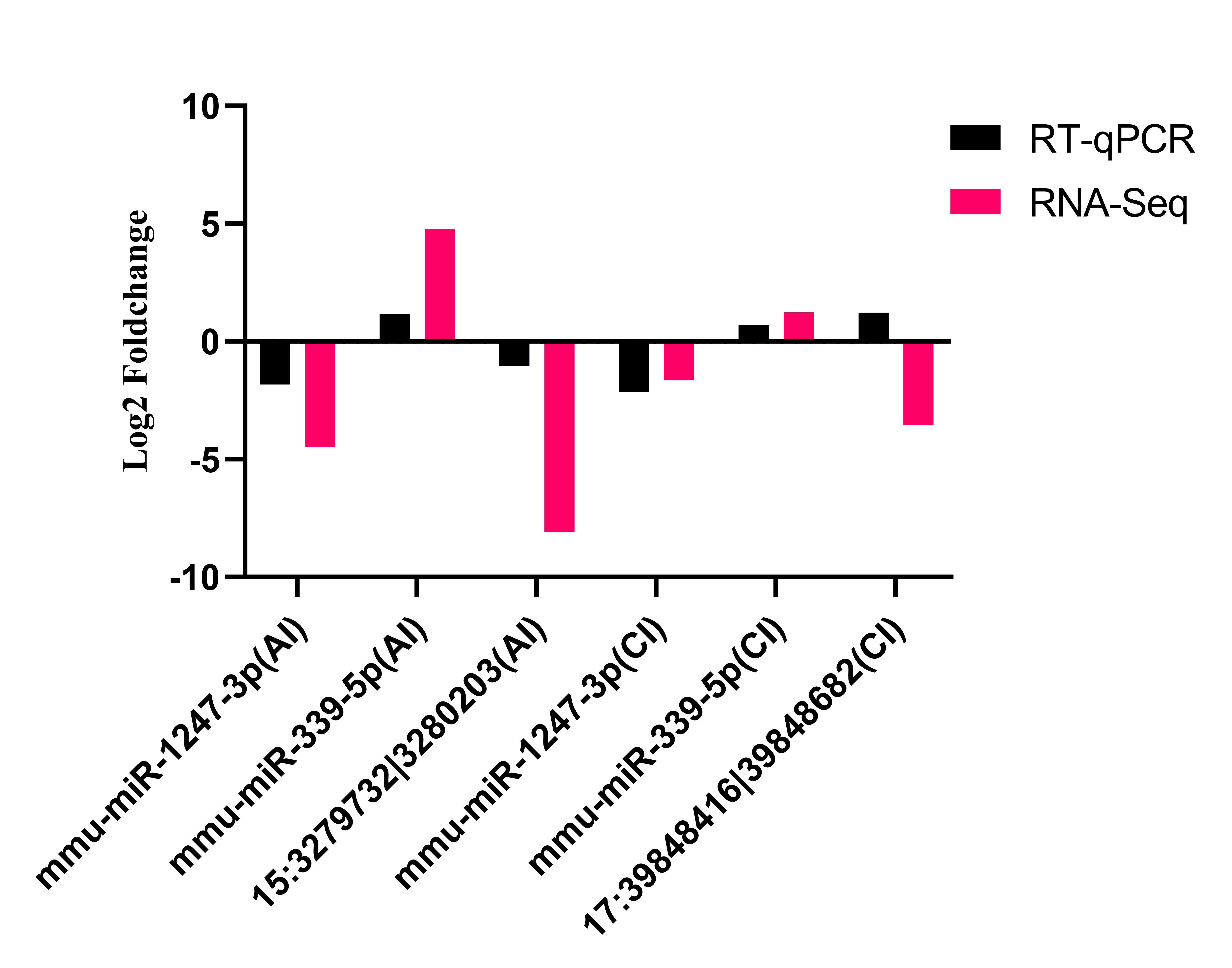

Supplement: Supplementary Figure 1 — Validation for the expression of the DEcircRNAs and DEmiRNAs by qRT-PCR at acute infection (AI) and chronic infection (CI) stages. [file Image_1.tif]
